# Supplementary material for: Content-rich biological network constructed by mining PubMed abstracts
Source: BMC Bioinformatics. 2004 Oct 8;5:147. doi: 10.1186/1471-2105-5-147 (PMC528731; doi:10.1186/1471-2105-5-147)
Supplement: Additional File 2 — The original results of the above study (non-essential files are deleted to keep the file size under the limit set by BMC bioinformatics). [file 1471-2105-5-147-S2.bz2 › chilibotAdditionalFile2/dip10/63ID7729427E222/html/TAF11_TBP.html]

 


 **TAF11** and **TBP** 
  
Found 10 abstracts in PubMed, retrieved 10.  
 

 What does Google say? 
 PDF only 
| .edu only 

---

**Interactive relationship** (e.g. stimulation, inhibition, etc)

**Inhibitory relationship**- Although it is well known that the TAF N terminal domain TAND at the amino terminus of the TAF1  [ **TAF11** ]  protein binds to  **TBP**  and thereby inhibits  **TBP**  function in vitro, the physiological role of this domain remains obscure.  Ref: 12582246 Nucleic Acids Res, 2003
**Neutral relationship**- Unexpectedly, these assembly experiments reveal that  **TAF11**  and TAF13 appear to provide the critical functional contacts with  **TBP**  during PIC assembly.  Ref: 12840001 EMBO J< MedlineTA>EMBO J, 2003
- In agreement with this, biochemical experiments indicate that a subpopulation of TAF7L is tightly associated with  **TBP**  in both pachytene and haploid cells and TAF7L interacts with the TFIID  [ **TBP** ]  subunit TAF1  [ **TAF11** ] .  Ref: 12665565 J Cell Sci, 2003
- **TBP**  recruitment was compromised in two different mot1 mutant strains, but was only moderately affected in a taf1  [ **TAF11** ]  Ts strain.  Ref: 12356733 EMBO J, 2002

**Non-interactive relationship** (e.g. studied together, co-existance, homology, etc.)

- The phenotypes included a low steady state level of the mutant  **TBP** , transcriptional derepression, dominant slow growth partial toxicity, and synthetic toxicity in combination with a deletion of the TAF1  [ **TAF11** ]  TAND domain.  Ref: 12697819 Mol Cell Biol, 2003
- Interestingly, a set of genes affected in the taf1  [ **TAF11** ]  DeltaTAND mutant is similarly affected in the taf12 HFD mutants but not in the nsl mutants of  **TBP** .  Ref: 12582246 Nucleic Acids Res, 2003
- In our previous study, we screened for mutations that cause lethality when co expressed with the TAF1  [ **TAF11** ]  gene lacking TAND taf1  [ **TAF11** ]  DeltaTAND and identified two DeltaTAND synthetic lethal nsl mutations as those in the SPT15 gene encoding  **TBP** .  Ref: 12582246 Nucleic Acids Res, 2003
- Our finding that recruitment of TFIID  [ **TBP** ] , by insertion of a  **TBP**  binding site upstream of the TDE1, restores basal but not activated transcription supports the model that TAF1  [ **TAF11** ]  carries out two independent functions at the cyclin D1 promoter.  Ref: 12569092 J Biol Chem, 2003
- Electrophoretic mobility shift assays reveal that binding of ts13 TAF1  [ **TAF11** ]  containing TFIID  [ **TBP** ]  complexes to the cyclin D1 TDE1 occurs at 25 degrees C but not at 37 degrees C in vitro and involves the initiator element.  Ref: 12569092 J Biol Chem, 2003
- Mutations in the histone fold domain of the TAF12 gene show synthetic lethality with the TAF1  [ **TAF11** ]  gene lacking the TAF N terminal domain TAND by different mechanisms from those in the SPT15 gene encoding the TATA box binding protein  [ **TBP** ]   **TBP** .  Ref: 12582246 Nucleic Acids Res, 2003
- The TAND domain of TAF1  [ **TAF11** ]  plays a primary inhibitory role at very few genes, but its function becomes widespread when other  **TBP**  interactions are compromised.  Ref: 12419230 Mol Cell, 2002
- As is evident from structural and functional studies,  **TBP**  s concave DNA binding surface is inhibited by a number of potential mechanisms, including homodimerization and binding to the TAND domain of the TFIID  [ **TBP** ]  subunit TAF1  [ **TAF11** ]  yTAF II 145 130.  Ref: 12697819 Mol Cell Biol, 2003
